# Severe asthma patient with secondary *Citrobacter koseri* abdominal infection: first case report and review of the literature

**DOI:** 10.1186/s13099-023-00574-9

**Published:** 2023-10-25

**Authors:** Mo Xian, Xiaolong Ji, Mingyu Zhong, Danhong Su, Jing Guan, Ruchong Chen

**Affiliations:** 1 State Key Laboratory of Respiratory Disease, National Clinical Research Center for Respiratory Disease, Guangzhou Institute of Respiratory Health, Department of Allergy and Clinical Immunology, The First Affiliated Hospital of Guangzhou Medical University, Guangzhou, 510120 Guangdong China; 2 Department of Laboratory Medicine, The First Affiliated Hospital of Guangzhou Medical University, Guangzhou, 510120 Guangdong China

**Keywords:** *Citrobacter koseri*, Severe asthma, Abdominal infection, Opportunistic pathogen

## Abstract

*Citrobacter koseri* (*C. koseri*) is a Gram-negative, motile, non-spore-forming facultative anaerobic bacillus belonging to the *Enterobacteriaceae* family. *C. koseri* typically utilizes citrate as the sole carbon source and constitutes part of the normal gastrointestinal flora in humans and animals. As an opportunistic pathogen, *C. koseri* infections are mainly observed in neonates, elderly individuals, and immunocompromised hosts. *C. koseri* has been one of the main etiological agents of neonatal meningitis and cerebral abscess. In recent years, an increasing number of cases have been reported in adults with severe infections caused by *C. koseri.* Here, we report for the first time a clinical case of concurrent *C. koseri* intra-abdominal infection in a patient with severe asthma and provide a brief review of the relevant literature. With this report, we hope to increase awareness and alertness among clinicians to the possibility of concurrent infection of gut commensal bacteria in asthmatic patients requiring long-term oral corticosteroid administration.

## Background

The genus *Citrobacter* belongs to the family *Enterobacteriaceae* and comprises different species of facultative anaerobe and motile, Gram-negative bacilli, which are oxidase negative and typically utilize citrate as the sole carbon source. Members of the genus *Citrobacter* are ubiquitous in nature, often found in water, food, and soil. Some of these bacilli constitute part of the microbial flora in the alimentary tract of humans and animals [1]. In 1993, Brenner et al. applied DNA hybridization to identify the genetic relatedness of 112 strains and concluded that the genus contains 11 genomospecies: *C. amalonaticus, C.farmeri, C. braakii, C. freundii, C. gillenii, C. murliniae, C. sedlakii, C. werkmanii, C. youngae, C. koseri, C. rodentium* [2]. It is recognized that *C. amalonaticus, C. braakii, C. koseri* (former *C. diversus*), *C. freundii* and *C. youngae* are pathogenic to humans [3]. They can cause infections in numerous organs, including the urinary, digestive, respiratory, and central nervous systems [3, 4]. The risk factors for *Citrobacter* infections remain obscure. As opportunistic pathogens, *Citrobacter* infections were predominantly observed in neonates, the elderly, and immunocompromised hosts, such as diabetics or those on immunosuppressive therapy [5, 6]. Since *C. koseri* exhibit neurophilic properties, they usually induce central nervous system abscesses in neonates and infants whose blood–brain barrier is immature [6]. Prior to the past decade, reports of *C. koseri* causing abscesses in adults were relatively rare; nevertheless, there have been an increasing number of adult infections, including healthy adults without underlying illnesses [7–9]. Herein, we, for the first time, reported a case of *C. koseri* extra-pulmonary infection in an adult patient with severe asthma.

## Case presentation

A 49-year-old female with a past medical history of severe asthma and osteoporosis presented to the Department of Allergy and Clinical Immunology with a 2-week history of arthralgia of the left hip and knee and a 6-day subjective fever, with a maximum body temperature of 39.6 °C. The patient had been on high-dose inhaled corticosteroids (ICS) fluticasone propionate and long-acting β2-agonist (LABA) formoterol fumarate (500 μg of fluticasone and 50 μg of formoterol twice daily) and low-dose (6 mg daily) oral corticosteroids (OCS) for more than 10 years, and she had previously undergone surgical treatment for lumbar osteoporosis combined with pathological fracture. Additionally, the patient denied any family medical history resembling her symptoms or other inherited disorders. At admission, the patient presented with a full moon face, buffalo hump, centripetal obesity, and stable vital signs. The physical examinations were grossly normal, except for percussion pain in the left hip. Routine laboratory tests were significant for elevated blood leukocyte count at 25.70 × 10^9^/L (4.0–10.0 × 10^9^/L), neutrophil count at 21.7 × 10^9^/L (1.8–8.0 × 10^9^/L), neutrophil ratio at 84.3% (40.0–70.0%), procalcitonin (PCT) at 0.19 ng/mL (0–0.05 ng/mL), and erythrocyte sedimentation rate (ESR) at 90 mm/h (0–20 mm/h). Peripheral T-lymphocyte subpopulation and absolute count manifested decreased levels of suppressor T-lymphocyte (TS), B-lymphocyte, and natural killer (NK) cells. Blood and sputum cultures for bacterial pathogens were all negative, as were serologies for common respiratory pathogens. X-rays revealed degeneration and osteoporosis of the left hip and the left ankle, while chest CT did not show any significant abnormalities.

The initial consideration was given to infection because of the patient's febrile symptoms and elevated inflammatory markers. Peripheral blood culture was obtained, and simultaneously, the patient was started on intravenous (i.v.) piperacillin-Sulbactam (3.0 g, Q8h) as the empirical anti-infective therapy. After 6 days of treatment, the patient was still feverish (temperature range between 38 and 39 ℃) and experiencing joint pain (mainly in the left hip, knee, and ankle), which may temporarily resolve after the fever had subsided. A multidisciplinary consultation was then convened, and the diagnostic possibilities of steroid-related osteoporosis, osteomyelitis, and infectious fever were discussed. In the meantime, *brucellosis* infection could not be excluded as the patient's clinical features were similar to the typical symptoms of *brucellosis* infection: undulant fevers, sweating, and migratory arthralgia and myalgia [10, 11]. While awaiting blood culture results, the anti-infective regimens were subsequently adjusted to i.v. meropenem (1 g, Q8h) and oral minocycline (100 mg, BID). After 5 days of adjusted treatment, the patient remained febrile, but her daily fever spikes showed a decreasing tendency (daily peak body temperature from 39.1℃ to 37.5℃). However, during this phase, the root cause of her fever was still not well known. Multiple blood and sputum bacterial cultures were negative, and magnetic resonance imaging of lower extremity joints revealed no evidence of osteomyelitis.

**Fig. 1:**
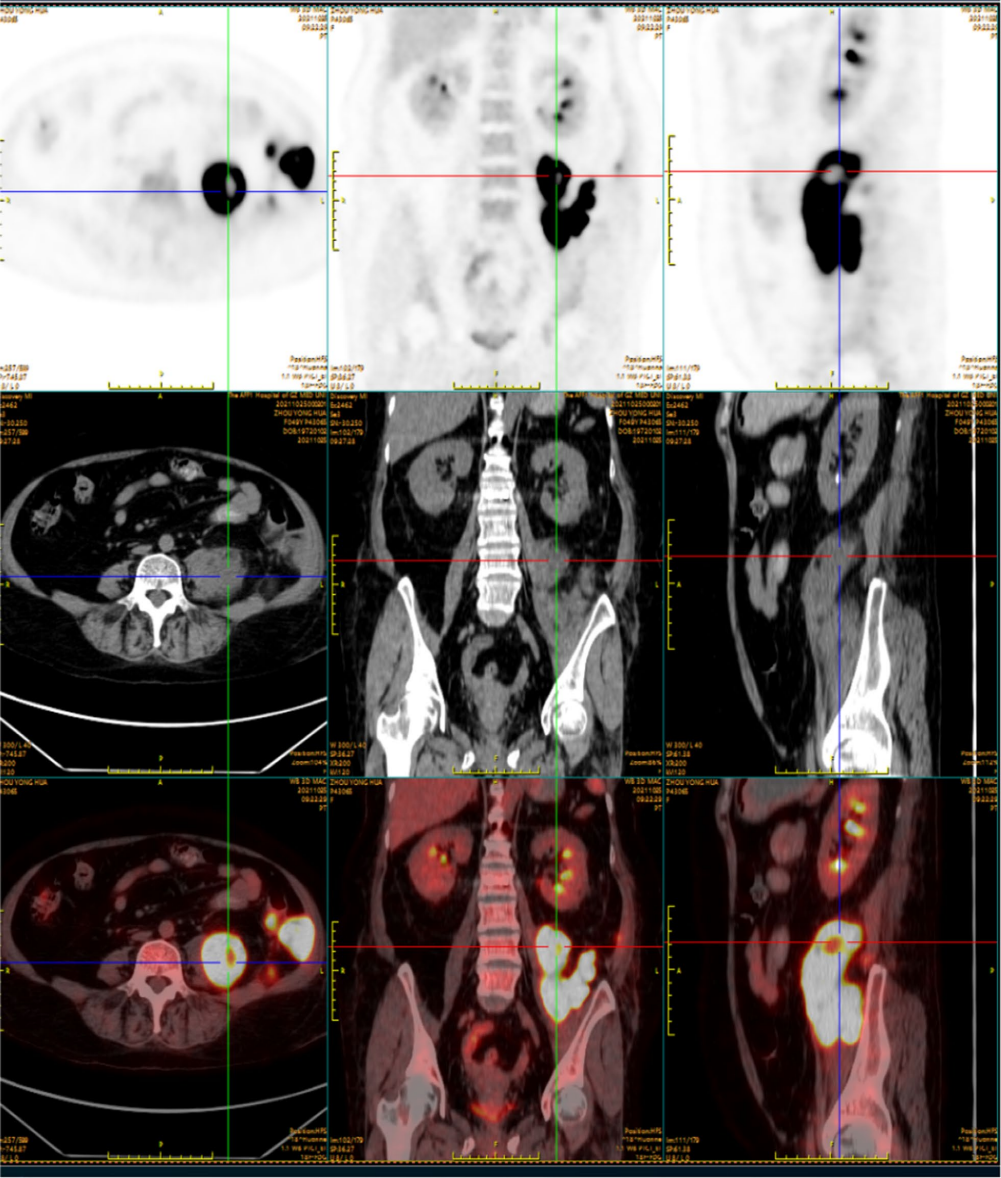
The coronal and sagittal section on abdominal ^18^F-FDG PET/CT. ^18^F-FDG PET, CT on the soft-tissue window, and fused PET/CT images demonstrated FDG accumulation in the left middle and lower abdomen. The hypermetabolic areas (yellow-white) indicated that the FDG-avid soft tissue mass was in an active inflammatory process

**Fig. 2:**
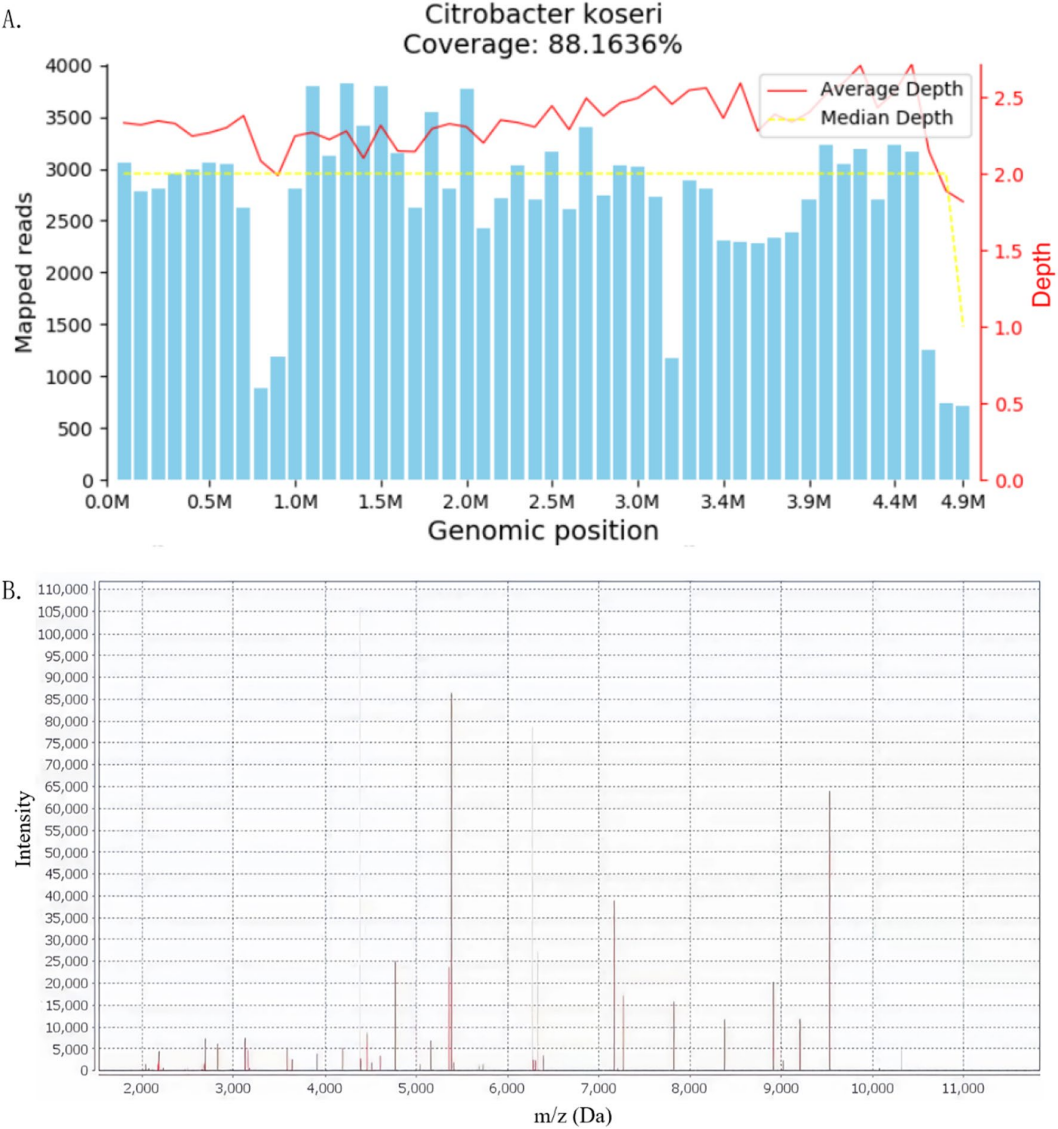
m-NGS **A** and mass spectrometry **B** analyses of abdominal biopsy tissue indicated *C. koseri* infection. **A**: The *C. koseri* genome sequences mapping by m-NGS. The result of mNGS showed 263387 reads corresponding to the C. koseri, with a coverage of 88.1636%. The X-axis represents the genome size of *C. koseri* and the Y-axis represents the number of sequences detected within different genomic segments. **B**: Mass spectrum of *C. koseri.* MALDI-TOF mass spectrometry showed the specific spectrum, which matched the registered pattern of *C. koseri*. The X-axis represents the mass-to-charge ratio (m/z) of different peptide fragment ions and the Y-axis represents the ion intensity. Peaks at specific m/z values indicate the presence of corresponding ions in the sample. The intensity of each peak reflects the abundance of the corresponding ion. *m-NGS* metagenomic next-generation sequencing, *MALDI-TOF mass spectrometry* matrix-assisted laser desorption ionization time-of-flight mass spectrometry

**Fig. 3:**
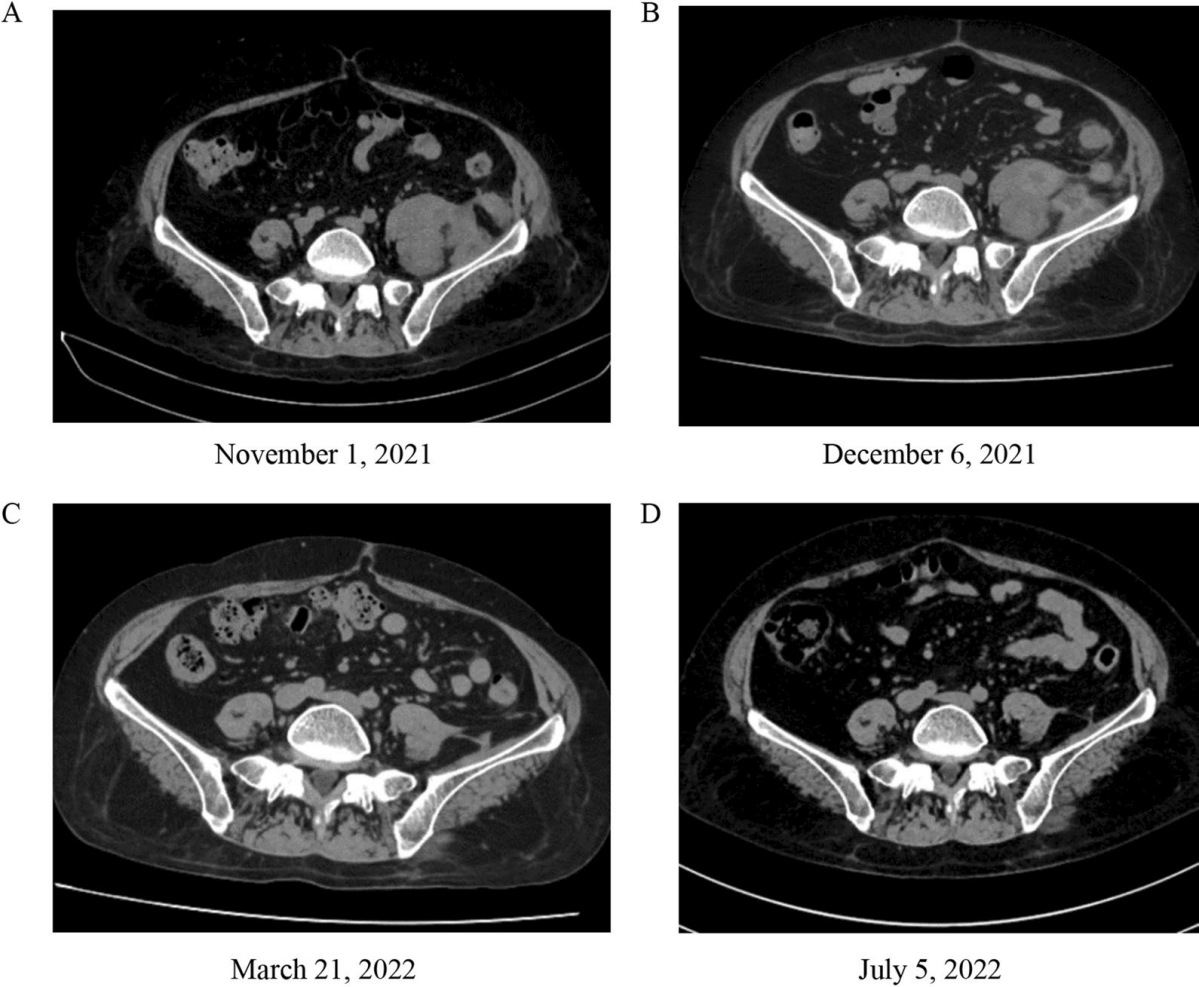
Comparison of the abdominal CT results before and after treatment. Serial CT scan images showed progressive resolution of the lesion in the left middle and lower abdomen over the course of 2 months on antimicrobial therapy: **A** prior to treatment; **B** 1 month after discharge; **C** 4 months after discharge; **D** 8 months after discharge

In order to identify the pathogen, conventional diagnostic tests and metagenomic next-generation sequencing (m-NGS) were performed simultaneously. m-NGS was performed with blood drawn at the peak of the febrile phase and detected *C. koseri* with 979 reads and a relative abundance of 38.36% (Table 1). Afterward, whole-body ^18^F-fluorodeoxyglucose positron emission tomography/computed tomography (^18^F-FDG PET/CT) was conducted to identify the infection foci. The PET/CT scan manifested a tissue-like mass with significantly increased FDG uptake in the left middle and lower abdomen. The delayed scan indicated a further increase in the lesion metabolism, suggesting a high possibility of infection while not ruling out the possibility of tumor (Fig. 1). The patient then underwent ultrasound-guided needle aspiration biopsy to define the nature of the FDG-avid lesion and to rule out the possibility of malignancy. The abdominal biopsy tissue was used for m-NGS, pathogen culture, and mass spectrometry (MS; Biomerieux Vitek, France). The m-NGS results of the biopsy tissue from the abdominal mass indicated *C. koseri* infection, with 263, 387 valid sequence reads and a coverage of 88.1636% (Fig. 2A, Table 1). This result echoes the m-NGS detection of blood sample. For pathogen culture, the colonies displayed a consistent morphology after inoculation, which was subsequently identified by mass spectrometry and also turned out to be *C. koseri* (Fig. 2B). Pathological analysis did not reveal tumor cells. In summary, *C. koseri* infection was confirmed by the mutual validation of blood m-NGS, biopsy tissue m-NGS, biopsy tissue culture, and mass spectrometry results. The antimicrobial susceptibility results indicated that the bacteria were susceptible to meropenem, ertapenem, ceftriaxone, amoxicillin-clavulanate, and levofloxacin. To explore the source of the infection, the patient subsequently received a colonoscopy, which did not demonstrate any structural abnormalities. After consulting the surgical department, conservative management was considered. Following confirmation of *C. koseri* infection, i.v. meropenem (1 g, Q8H) was continued to use as targeted antimicrobial therapy, while minocycline was discontinued. After receiving 12 days of continuous medication, the patient's fever subsided, and her arthralgia was also alleviated. The patient was discharged from the hospital and continued to be treated orally with faropenem (0.3 g, Q8h) for 2 months. During the 8-month follow-up, abdominal CT showed gradual lesion absorption (Fig. 3).

**Table 1:** Pathogens detected by microbiological culture and m-NGS

Sample type	Microbiological culture	m-NGS^a^		
		Species	Reads	Relative abundance (%)
Plasma^b^	Negative	*Citrobacter koseri*	979	38.36
Abdominal biopsy tissue	*Citrobacter koseri*	*Citrobacter koseri*	263, 387	78.30

^a^Cell-free DNA was used for mNGS

^b^From peripheral blood drawn in the febrile phase

## Discussion

Due to chronic airway inflammation, patients with severe asthma need to inhale large amounts of ICS or even take OCS for an extended period to control their asthma symptoms. Howbeit, long-term administration of oral corticosteroids may induce many detrimental effects on metabolism, bone density, and especially immunity [12]. Our case manifested decreased amount of TS-lymphocyte, B-lymphocyte, and NK cells, suggesting that host immunosuppression due to anti-asthmatic drugs may be a predisposing factor in the *C. koseri* infection. With the exception of the patient’s comorbidities, the infection foci and pathogenic bacteria of our case were similar to those reported by Yamamoto et al. and Lin et al. [13, 14]. To some extent, normal colonoscopy results had excluded the possibility of abdominal infection secondary to intestinal perforation. Therefore, the formation of abdominal *C. koseri* foci is potentially attributable to insidious hematogenous or lymphatic dissemination. Since the lesion is located along the path of the femoral nerve, nerve compression might partially explain the patient's lower extremity pain after the suspicion of osteomyelitis had been excluded.

Since there are no characteristic lesions of *C. koseri* infection, and its colonies are easily confounded with bacteria of the family *Enterobacteriaceae*, especially *Escherichia coli*, it is reluctant to reliably diagnose *C. koseri* infection based solely on clinical manifestations. The diagnosis primarily relies on the isolation and identification of *C. koseri*. At present, bacterial culture, 16S rRNA gene PCR amplification, and m-NGS are well-established and complementary methods for confirming the diagnosis. m-NGS is a high-throughput or massively parallel sequencing method capable of detecting a wide variety of pathogens, including bacteria, viruses, fungi, and parasites [15]. Compared to traditional blood cultures, mNGS significantly increases the diagnostic sensitivity of the patient’s pathogens even after antibiotic treatment, while blood cultures are often negative [16, 17]. In this case, multiple blood cultures had failed to identify the pathogen. *Citrobacter spp.* was detected with the help of plasma m-NGS, and the test results were corroborated with the tissue biopsy outcomes. Taking this evidence together, our case suggested that the m-NGS test is competent to identify potential pathogens missed by conventional detection methods within a relatively short period (24 h) and can serve as a conducive complement in detecting infectious fever.

In general, most *Citrobacter* infections are treated with antibiotics. However, there are no comparative studies on antibiotic treatment of *Citrobacter* infection. Based on antimicrobial susceptibilities, carbapenems or quinolones appear to be the preferred therapeutic options for *C. koseri* infection [5]. Aminoglycosides or third-or-fourth-generation cephalosporins are also therapeutically effective, but some strains of *C. koseri* may present resistance to these two classes of antibiotics [18]. To date, there are no established treatment guidelines for *Citrobacter* infections. In addition, due to the rarity and severity of *C. koseri* infection, it is difficult to determine the optimal dose and treatment duration through prospective clinical studies. The therapeutic regimens are formulated chiefly based on retrospective investigations, case reports, and expert opinions [5, 14, 19]. As the drug susceptibility of different strains may differ considerably, the selection of antimicrobial treatment should be on the basis of drug susceptibility testing. Moreover, the selection of antimicrobials should also be based on the site of infection. Criteria for discontinuation of antibiotics include alleviation of clinical symptoms and resumption of behavioral and biochemical indicators.

In summary, we reported an unusual case of abdominal infection caused by *C. koseri* in a patient with long-term steroid application due to severe asthma. The identification of *C. koseri* was achieved by m-NGS and subsequently confirmed by pathogen culture and mass spectrometry of the biopsy samples collected from the abdominal lesion. Both the patient’s clinical symptoms and the abdominal lesion gradually resolved after carbapenem therapy. Clinicians need to be vigilant regarding the possible *Citrobacter* infection in patients with severe asthma or the long-term application of corticosteroids.

## Data Availability

Not applicable.
